# The evolution of American College of Surgeons alcohol screening and brief intervention mandates

**DOI:** 10.1186/1940-0640-7-S1-A4

**Published:** 2012-10-09

**Authors:** Douglas Zatzick, Larry Gentillelo, Gregory Jurkovich, Dennis Donovan, Chris Dunn, Rick Ries, Frederick Rivara, Daniel Hungerford

**Affiliations:** 1 Harborview Injury Prevention and Research Center, University of Washington School of Medicine, Seattle, WA, USA; 2 Southwestern Medical Center, University of Texas, Dallas, TX, USA; 3 National Center for Injury Prevention and Control, Centers for Disease Control and Prevention, Atlanta, GA, USA

## 

Over the past five years, trauma-center alcohol screening and brief intervention (SBI) implementation has advanced considerably. A key catalyst has been the willingness of the American College of Surgeons Committee on Trauma (ACS/COT) to consider policy guidelines based on empiric investigation. We summarized data on and policy discussions pertaining to the college’s current implementation of the alcohol SBI mandate at US trauma centers. Trauma programs at all US level-I trauma centers (N = 204) were contacted and asked to complete a survey regarding alcohol SBI practices in the year before the ACS/COT requirement became standard practice. A questionnaire that assessed alcohol screening methods and intervention capacity was developed to evaluate premandate SBI practices. Of the 204 level-1 trauma centers contacted, 148 (73%) responded to the survey. Over 70% of responding centers routinely employed laboratory tests to screen patients for alcohol; 39% routinely used a screening questionnaire or standardized screening instrument. Patients who screen positive for an alcohol use disorder (AUD) receive a formal alcohol consult or an informal alcohol discussion with a staff member approximately 25% of the time. We conclude that there was marked variability across level-I trauma centers in the percentage of patients screened for AUD and in the nature and extent of intervention among those who screened positive before implementation of the ACS/COT alcohol SBI mandate. In the wake of the mandate, orchestrated research and policy efforts could systematically implement and evaluate training in the delivery of evidence-based SBI as well as training in the development of trauma-center organizational capacity for the sustained delivery of SBI.

